# ToggleMimic: A Two-Stage Policy for Text-Driven Humanoid Whole-Body Control

**DOI:** 10.3390/s25237259

**Published:** 2025-11-28

**Authors:** Weifeng Zheng, Shigang Wang, Bohua Qian

**Affiliations:** School of Automation, Guangxi University of Science and Technology, Liuzhou 545006, China; 20230202049@stdmail.gxust.edu.cn (W.Z.); 20230203011@stdmail.gxust.edu.cn (B.Q.)

**Keywords:** humanoid, imitation learning, learning-based control, policy distillation

## Abstract

For humanoid robots to interact naturally with humans and seamlessly integrate into daily life, natural language serves as an essential communication medium. While recent advances in imitation learning have enabled robots to acquire complex motions through expert demonstration, traditional approaches often rely on rigid task specifications or single-modal inputs, limiting their ability to interpret high-level semantic instructions (e.g., natural language commands) or dynamically switch between actions. Directly translating natural language into executable control commands remains a significant challenge. To address this, we propose ToggleMimic, an end-to-end imitation learning framework that generates robotic motions from textual instructions, enabling language-driven multi-task control. In contrast to end-to-end methods that struggle with generalization or single-action models that lack flexibility, our ToggleMimic framework uniquely combines the following: (1) a two-stage policy distillation that efficiently bridges the sim-to-real gap, (2) a lightweight cross-attention mechanism for interpretable text-to-action mapping, and (3) a gating network that enhances robustness to linguistic variations. Extensive simulation and real-world experiments demonstrate the framework’s effectiveness, generalization capability, and robust text-guided control performance. This work establishes an efficient, interpretable, and scalable learning paradigm for cross-modal semantic-driven autonomous robot control.

## 1. Introduction

In recent years, deep reinforcement learning (DRL) and imitation learning have made significant progress in the field of robotic control, enabling more precise motion execution and improving cross-task generalization capabilities. Nevertheless, allowing robots to flexibly understand and adapt to human instructions in multi-task scenarios remains challenging. Traditional state-action learning models often bind policies to specific task distributions, making it difficult to dynamically switch control behaviors based on changes in goals or contextual instructions. Moreover, expert-level visuomotor policies tend to incur high computational costs and exhibit weak transferability when jointly modeling high-dimensional states with natural language inputs, limiting the practical reuse of learned skills during deployment.

Natural language provides an intuitive and scalable interface for human–machine interaction. Large-scale pre-trained language models [1,2] have demonstrated remarkable capabilities in capturing semantic structures and human intentions [3]. However, there remains a significant semantic gap between abstract language representations and low-level motion control signals. Directly mapping language embeddings to the action space can easily lead to unstable policy learning and cause misalignment between user intent and executed actions.

Recent research has made progress in narrowing this gap by integrating cross-modal alignment into policy learning and promoting synergistic reasoning between language and physical representations. These approaches demonstrate that anchoring language to a sensory-motor latent space can enhance semantic consistency and control reliability [4]. However, existing methods still exhibit limitations in terms of efficient skill transfer, deployment costs, and scalable adaptability to new instructions.

To address these limitations in semantic alignment and deployment efficiency, we introduce ToggleMimic, enabling task-agnostic action switching through natural language commands. The framework employs a two-phase architecture.

### 1.1. Fused Attention Policy (FAP)

The teacher policy (Fused Attention Policy, FAP) is trained via Proximal Policy Optimization (PPO)-based imitation learning to minimize the divergence between FAP-generated actions and expert trajectories. This objective necessitates three core modules: (1) Early Fusion Layer (EF)—integrates text embeding $v_{t}$ and robot state $S_{t}$ to construct a text-augmented state representation $H_{t}$; (2) Observation feature extraction module (μ module)—projects $H_{t}$ onto state-semantic representation $O_{t}$ for downstream processing; (3) LightCrossAttention (LCA)—jointly models state-semantic representation $O_{t}$ and text embeddings $v_{t}$ via cross-attention, enabling context-aware action adaptation to natural language commands; (4) Action Generation Layer (AGL)—the outputs from the μ module $O_{t}$ and the LightCrossAttention module *Y* are fused and then mapped to the robot’s action space by a Multilayer Perceptron (MLP).

Crucially, FAP’s training objective is to regulate the conditional action distribution, conditioned on natural language inputs rather than labeled text. As a text-conditioned expert policy, FAP provides a reproducible distribution of language-grounded demonstrations for student distillation in Stage 2.

### 1.2. Student Distilled Policy (SDP)

The student policy adopts a highly analogous architecture to the FAP’s backbone, enabling efficient knowledge distillation. This architectural parity preserves the teacher’s semantic-action fusion capability (specifically the LCA module’s cross-semantic alignment), allowing the student to inherit robust semantic grounding without architectural interference. To enhance distributional fidelity and training robustness, we introduce two FAP-extended modules: (1) Gating Network—dynamically modulates the fusion strength between state-semantic representation $O_{t}$ and text embeddings $v_{t}$ via learnable gating weights; (2) Action Classification Head—through multi-task learning, this improves both the consistency and discriminability of action representations.

The loss function for SDP combines behavioral cloning $L_{\mathrm{BC}}$ and bidirectional KL divergence $L_{\mathrm{KL}}^{bi}$, enabling direct action mapping that preserves the teacher’s cross-semantic alignment capability through unified neural architecture and loss design.

Our method enables language-instructed semantic-action mapping via teacher-student imitation learning, preserving cross-task alignment fidelity through the LCA module.

In summary, the main contributions of this paper can be summarized as follows:

1. We propose a text-driven two-stage imitation learning framework, ToggleMimic, which enables action-switching control under language conditions.
2. We design a cross-modal fusion teacher policy network that combines text and robot states using a LightCrossAttention, enhancing the language-to-action correspondence strategy.
3. We construct a student policy network with Gating Network and Action Classification Head, significantly improving model generalization and reducing the difficulty of deployment in real-world scenarios.

The structure of this paper is as follows. Section 2 reviews related work on humanoid control and language-driven motion generation. Section 3 details our ToggleMimic methodology, including the two-stage policy design and distillation process. Section 4 presents experimental results and analysis. Section 5 provides the discussion and limits.

## 2. Related Work

### 2.1. Learning-Based Whole-Body Control for Humanoid Robots

Early approaches to humanoid robot motion control relied heavily on dynamics modeling, utilizing algorithms such as Zero Moment Point (ZMP) or Model Predictive Control (MPC) to manage gait control [5,6]. However, due to the high dimensionality of humanoid robot systems, designing such controllers was both time-consuming and labor-intensive. Moreover, these methods often struggled with simple states and frequently failed in complex scenarios. With the advent of domain randomization [7] and the advancement of GPU parallel computing [8,9], constructing whole-body controllers for humanoid robots using reinforcement learning or imitation learning algorithms has become mainstream. These algorithms are referred to as learning-based humanoid robot motion control.

Currently, there are two main approaches to achieving learning-based humanoid robot motion control. The first involves designing sophisticated reward functions to guide robots in executing relevant motions, while the second leverages datasets such as AMASS to achieve whole-body control by tracking motions present in those datasets. Prior research has demonstrated that pure reward-function-guided reinforcement learning can enable different types of robots—such as quadrupeds and humanoids—to perform basic functions like walking and running [10,11,12,13,14,15]. There are also specialized controllers that design reward functions for specific tasks, achieving unique motor skills such as fall recovery [16,17] or dynamic getting-up [18,19]. However, as noted earlier, reinforcement learning-based humanoid robot controllers designed purely with reward functions often only excel in specially tailored tasks and struggle to generalize to untrained scenarios. For instance, a skill designed for jumping may fail to generalize to running. This necessitates the individual design of reward functions for each task, with more complex actions requiring increasingly intricate rewards, thereby constraining the robot’s versatility.

Using datasets to train robots via imitation learning represents an alternative approach to circumventing the design of complex reward functions. Recent studies have highlighted several high-quality datasets featuring physically plausible human motion tracking [20,21,22]. Most existing work transforms motion control problems into dataset-tracking problems, leveraging these datasets to develop high-performance motion controllers. Representative work includes ASAP [23], which employs a two-stage network for dataset tracking training and utilizes an incremental action model to achieve agile motion execution; and KungfuBot [24], which also uses a two-stage network and introduces adaptive tracking mechanisms to enhance tracking performance, enabling complex movements like kung fu and dance. While both ASAP and KungfuBot demonstrate agile tracking for individual motions, their limitation lies in single-motion alignment—one model corresponds to one action—which restricts the generalizability of these models across diverse tasks.

### 2.2. Motion Generation

Early research on motion generation tended to rely on high-level neural networks to output motion trajectories, which were then tracked and executed by low-level controllers. DeepMimic [25] enables characters to imitate diverse motions, AMP [26] using adversarial imitation learning to tracking motions, HumanPlus [27] trains a high-level policy through behavioral cloning using multimodal data collected from human teleoperation. By integrating visual input and proprioceptive feedback, it outputs target pose sequences to complete downstream tasks. MDM [28]/PriorMDM [29]/ReMoDiffuse [30] employ diffusion models to generate motion sequences from textual inputs. OmniH2O [31] uses a pre-trained diffusion model to produce fixed-length trajectories and then trains a low-level controller via reinforcement learning to achieve whole-body tracking, enabling real-world deployment. Exbody [32]/Exbody2 [33] utilizes a c-VAE model, where subsequent motions are generated by a Transformer given historical motion data.

Some studies have incorporated physical constraints into motion generation to prevent issues like uncontrolled movements. For example, GMD [34] applies projections to the motion generation process guided by text; RobotMDM [35] adds Q-function constraints, both achieving a degree of physically plausible human motion generation. HumanMimic [36] applies a specific Integral Probabilistic Metric which can stabilize the training process and prevent mode collapse.

To bridge the gap between high-level trajectories and low-level control, end-to-end motion generation has become a research hotspot. DiffuseLoco [37] employs a diffusion model to achieve unified control of agile motion skills for quadruped and biped robots. BeyondMimic [38] uses a guided-diffusion-based unified framework to achieve high-quality motion tracking. However, due to the high degrees of freedom in robots—particularly the complexity of their high-dimensional dynamics—the direct cross-modal generation of motions remains an area ripe for breakthroughs.

### 2.3. Cross-Modal Language-Action Policies

Translating natural language instructions into robot action sequences is key to achieving natural human–machine interaction. Some approaches separate high-level planning from low-level control. For instance, SayCan [39] uses LLMs to assess task feasibility and generate high-level skill sequences for execution by low-level policies.The reliability of such pipelines benefits from recent advancements in the field of data quality assurance, such as VLSR [40] and VDC [41], which both utilize LLMs to clean and cluster datasets. Works like VIMA [42] and RT-2 [43] explore an end-to-end paradigm that jointly embeds language, vision, and actions into a single model, demonstrating strong generalization capabilities. For humanoid robots, LangWBC [44] proposes an end-to-end framework that uses language instructions to guide whole-body control. Beyond linguistic interfaces, recent human–robot communication frameworks [45] provide structured gesture design methodologies for warehouse environments, enriching the multimodal interaction landscape.

Compared with the aforementioned works, our ToggleMimic framework focuses on learning language-conditioned skills from high-quality human motion data (AMASS) and transforms a privileged-information-dependent teacher policy into a student policy requiring only proprioceptive and textual inputs through a carefully designed two-stage distillation process. This achieves efficient, diverse, language-driven control on low-cost computing platforms.

## 3. Methods

**Figure 1:**
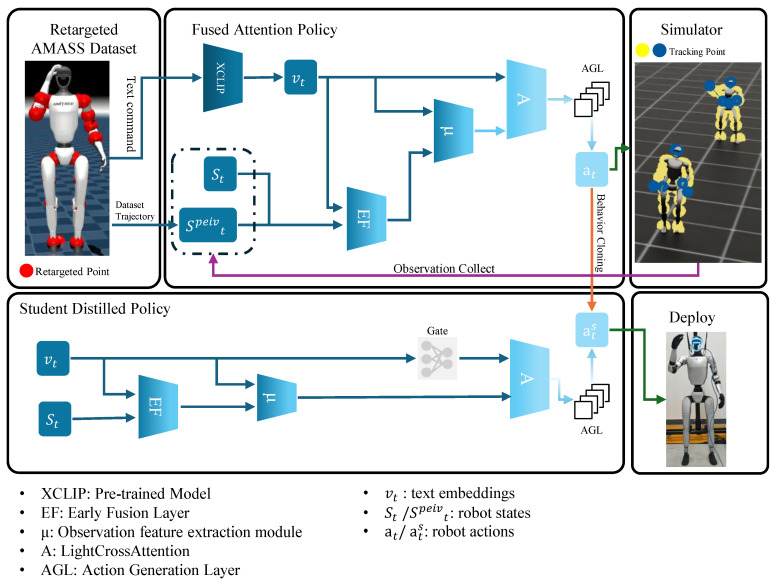
ToggleMimic framework. The training process of ToggleMimic consists of a semantic and motion tracking teacher training phase and a distillation training phase. We first re-target the motion capture dataset and use reinforcement learning to train the teacher policy with semantics based on a cross-attention architecture. Subsequently, we distill away the privileged data from the teacher policy using a gating module and loss function. During deployment, we utilize the student policy on an i7 8th generation processor to achieve zero-shot sim-to-sim and sim-to-real, demonstrating that our method is capable of generating diverse text-based actions.

We propose ToggleMimic, an end-to-end imitation learning framework that generates robotic motions from textual instructions, enabling language-driven multi-task control. The algorithmic framework is illustrated in Figure 1. The teacher policy (Fused Attention Policy, FAP) is trained using Proximal Policy Optimization (PPO) [46], utilizing proprioceptive, privileged data (including friction, dataset trajectory, etc.), and text labels as observation data. The teacher policy trains an action generation model incorporating privileged data, which serves as the expert strategy for high-quality action generation in the second stage.

The distillation process explicitly operates without privileged data, producing a policy requiring only text labels and robot state. This Student Distilled Policy (SDP) enables effective reasoning in real-world deployment through its minimal-input design.

### 3.1. Dataset and Preprocessing

#### 3.1.1. Choice of AMASS Dataset

Our method is trained and evaluated on a curated subset of the AMASS dataset [20]. AMASS was chosen for this work due to its unparalleled scale and diversity as a large-scale, unified human motion database. It aggregates multiple high-quality optical marker-based motion capture (mo-cap) datasets, providing a vast collection of human activities ranging from basic locomotion to complex interactive actions. This diversity is crucial for training a general-purpose policy that can respond to a wide variety of natural language commands. Furthermore, AMASS provides motions represented by SMPL [47] model, which facilitates a standardized and robust data processing pipeline.

#### 3.1.2. Data Curation and Text–Action Pairing

We constructed a high-quality dataset for text-driven control using the ACCAD and BMLmovi subsets from AMASS, selected for their rich variety of structured whole-body motions with descriptive annotations. These annotations exhibit exceptional semantic density—each description is concise, purpose-driven, and devoid of superfluous content, ensuring every token carries meaningful information for motion specification. This high signal-to-noise ratio eliminates the need for models to filter irrelevant verbal filler during learning. We systematically aligned motion sequences with their textual descriptions to form (text, motion) pairs, with manual verification to ensure clarity, precision, and direct correspondence with each motion segment.

#### 3.1.3. Motion Re-Targeting to Humanoid Robot

The human motions in AMASS are not directly executable on a humanoid robot like the Unitree G1 due to differences in kinematics and dynamics. Therefore, we performed inverse kinematics (IK) motion re-targeting to transfer the human motions to the robot’s kinematic structure. We employed the Levenberg-Marquardt (LM) algorithm [48] to solve the IK problem, optimizing the robot’s joint angles to match the trajectories of key body positions (e.g., hands, feet, root) from the AMASS data. A smoothness constraint was incorporated into the optimization to eliminate jittering in the resulting joint trajectories, ensuring the generated motions are physically feasible and stable for the robot to execute.

(1) $$\min_{\theta_{t}}\sum_{t}∥f\left(\theta_{t}\right)-p_{t}^{mocap}{∥}^{2}+\lambda_{s}{∥\theta_{t}-\theta_{t-1}∥}^{2}$$

where $\theta_{t}$ are robot joint angles at frame *t*, f(·) maps joint angles to Cartesian positions, $p_{t}^{\mathrm{mocap}}$ are target positions from AMASS data, $\lambda_{s}$ is the smoothness weight, and *t* indexes time frames.

The outcome of this process is a set of kinematically feasible robot motion sequences that retain the semantic essence of the original human motions while being fully compatible with the Unitree G1’s mechanical constraints.

### 3.2. Fused Attention Policy (FAP)

Our Fused Attention Policy (FAP) trains a strategy that tracks motion using motion trajectories from AMASS dataset, while enabling action switching via text commands. The FAP policy’s input is divided into two components: (1) a combined observation of proprioceptive and privileged data; (2) 512-dimensional text embeddings derived from the pre-trained XCLIP model.

(2) $$v_{t}=f_{\mathrm{CLIP}}\left(c_{t}^{\mathrm{text}}\right)\in R^{512}$$

These two components jointly enable the robot to align its motion trajectory with text commands. The input structure for FAP’s observations is detailed in Table 1.

Our FAP network features three core designs: the Early Fusion (EF) layer, the Observation feature extraction module (μ module), and the LightCrossAttention module (LCA module).

(1) Early Fusion (EF) layer

The Early Fusion (EF) layer fuses the normalized observation with the text embedding via an MLP, producing a text-augmented state representation that modulates the proprioceptive using text semantics.

(3) $$H_{t}=\mathrm{MLP}\left(v_{t};S_{t};S_{t}^{priv}\right)$$

where $v_{t}$—text embedding; $S_{t}$—proprioceptive observation; $S_{t}^{priv}$—privileged observation.

The EF layer provides contextual constraints for the distribution of state features under different task instructions, enabling the network to dynamically adjust the state encoding strategy based on textual semantics. For instance, for instructions with significant semantic differences such as “raising a hand”, the fused features will vary along different subspace directions. This provides differentiated semantic guidance for subsequent motion feature extraction and cross-modal attention mechanisms. This low-level conditional feature fusion mechanism introduces semantically relevant constraints at the state feature level, facilitating the generation of more stable and semantically consistent motion trajectories by the model during language-driven action transitions.

(2) Observation feature extraction module (μ module)

**Figure 2:**
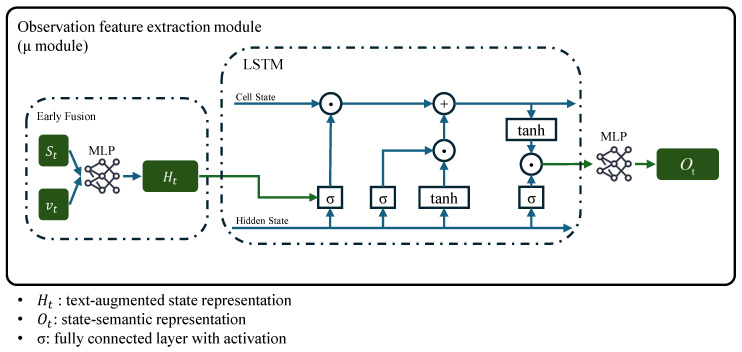
μ module. The μ module employs a hybrid LSTM-MLP structure to extract the state-semantic representation $O_{t}$ from the text-augmented state representation $H_{t}$. The LSTM captures temporal dependencies in the motion sequence, while the MLP extracts nonlinear features, ensuring a balanced representation for downstream action generation.

The Observation feature extraction module (Figure 2) is a core component of the FAP network, responsible for extracting state-semantic representation from the text-augmented state representation that can be used for action generation.

(4) $$O_{t}=M\left(L(H_{t},h_{t-1},c_{t-1})\right)$$

where L—LSTM, and M—a two-layer ReLU-MLP.

To capture temporal continuity in robotic motion while extracting nonlinear features from text-augmented state representation (generated in the EA layer), the μ module employs a hybrid LSTM-MLP structure. This design enables simultaneous nonlinear feature extraction from current observations (via MLP) and temporal memory for historical state sequences (via LSTM), ensuring temporal coherence across the motion trajectory. Consequently, the policy network achieves balanced local responsiveness and global dynamics during motion generation, producing stable state-semantic representations for subsequent multimodal action generation.

(3) LightCrossAttention (LCA)

**Figure 3:**
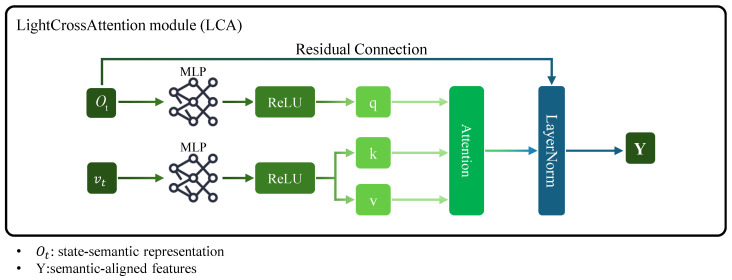
LightCrossAttention module. The LCA module aligns text embeddings $v_{t}$ with the state-semantic representation $O_{t}$. It computes semantic correspondence via cross-attention and uses residual connections with layer normalization to stabilize training, outputting semantic-aligned features *Y* for context-aware action generation.

To enhance model interpretability for text-guided robotic actions, we introduce LightCrossAttention (LCA) (Figure 3), a lightweight cross-modal attention module. LCA adopts a cross-attention structure that learns semantic-action correspondence, allowing the policy network to generate actions directly conditioned on linguistic instructions. By incorporating residual fusion and linear mapping, LCA maintains the stability of representation learning while minimizing computational overhead, enabling efficient deployment on resource-constrained robotic systems.

Given the state-semantic representation and text embeddings, the LCA module aligns the text features to the state representation space through a learned mapping, enabling semantic correspondence between language and sensory input.

(5) $$q=\sigma (W_{o}O_{t}),k=\sigma (W_{t}v_{t}),v=\sigma \left(W_{t}v_{t}\right)$$

(6) $$A=\mathrm{Attention}\left(q,k,v\right)=\mathrm{softmax}\left(\frac{qk^{T}}{\sqrt{d_{h}}}\right)v$$

(7) H=LayerNorm(q+A)

(8) $$Y=W_{o}^{\prime }H$$

where σ(·) is the ReLU activation function, $W_{o}$ and $W_{t}$ are linear projection matrices, $d_{h}$ represents the dimension of the hidden space, and *Y* represents the output semantic-aligned features.

The multi-head attention computes the association weights between the state-semantic representation and the text embedding, and the output attention result *A* represents the weighted semantic response of the state-semantic representation feature at each time step in the text embedding vector space. To enhance stability, residual connections and layer normalization are used for fusion. Finally, a linear mapping transforms the fused cross-modal features into semantic-aligned features.

(4) Action Generation Layer

After completing the cross-modal feature interaction, the model fuses the state-semantic representation features with the semantic-aligned features.

(9) $$f_{\mathrm{final}}=O_{t}+Y$$

where $O_{t}$—state-semantic representation and *Y*—semantic-aligned features.

This element-wise additive fusion retains the continuous dynamic information of the two types of state features, enabling the model to simultaneously consider the semantic constraints of the robot’s current state and the textual instruction during the action generation phase. The fused features, $f_{\mathrm{final}}$, are then fed into the Action Head, which maps the high-dimensional semantic features to the robot’s action space via an MLP:

(10) $$a_{t}=M\left(f_{\mathrm{final}}\right)$$

where $a_{t}\in R^{23}$—robot action space.

This structure achieves the fusion of state-semantic representation and semantic-aligned features, directly mapping them to the action space. It enables the model to generate control commands that comply with both task semantics and kinematic constraints, under the guidance of a joint semantic representation.

To accelerate the teacher policy’s learning from the dataset, our FAP strategy is trained using PPO algorithms. The reward function and its weights are shown in Table 2.

### 3.3. Student Distilled Policy (SDP)

Our Student Distilled Policy (SDP) replaces privileged inputs in the Fused Attention Policy, eliminating the need for inaccessible observations during deployment. To accelerate distillation efficiency, SDP preserves the FAP’s core network architecture. By inheriting this structure, the student avoids redundant learning of sensor-to-state mappings and instead focuses on reconstructing the decision-making features and control distributions of the Teacher within a constrained network. This significantly improves distillation stability and convergence speed. Crucially, we incorporate three domain-specific enhancements for robust generalization:

(1) Gating Network

To maintain semantically consistent control features after input data compression, the SDP introduces a Gating Network module after the Action Generation Layer. The state-semantic representation $O_{t}$, obtained through feature extraction, and the semantic-alignment feature *Y* are combined by computing fusion weights using a gating unit.

(11) $$G_{t}=O_{t}+\sigma \left(W_{g}\left[O_{t};Y\right]+b_{g}\right)⊙Y$$

where σ()—Sigmoid function and ⊙—element-wise multiplication.

The core mechanism employs learnable gating parameters to dynamically modulate the fusion of state-semantic representation and semantic-aligned features in the SDP. This enables the network to autonomously adjust the dependency strength between textual representations and proprioceptive across varying conditions. The gating structure allows the SDP to adaptively learn feature dependency dynamics during training—with robustness to observation noise or representation redundancy—ensuring action stability. Furthermore, the gating mechanism enhances SDP’s capacity for novel textual input recognition, strengthening task-switching capability and generalization across diverse operational scenarios.

(2) Action Classification Head (ACH)

To enhance distributional stability and action-space consistency in the student policy during action generation, we introduce an Action Classification Head (ACH) after the action distribution output of the Action Generation Layer. This module enables the model to distinguish between action categories aligned with instructional text while generating action distributions, thereby achieving separated boundary representations in the latent action space.

(12) $$z_{t}=W_{c}a_{t}^{s}+b_{c}$$

where $W_{c}$: the classification weight matrix, $a_{t}^{s}$: the action output distribution (SDP), $b_{c}$: the number of action categories.

Meanwhile, the action classification auxiliary head employs Cross-Entropy Loss:

(13) $$L_{ach}=\mathrm{CrossEntropy}\left(z_{t},y_{t}\right)$$

where $y_{t}$: action class label (one-hot encoded) at time *t*, and $z_{t}$: predicted probability.

Our Action Classification Head explicitly constrains the policy network to maintain distributional stability within the shared latent action space, making the distributions of similar actions more concentrated. It provides action-space-aligned gradient supervision during optimization, thereby enhancing generalization across instructional text and distributional stability in the initial training phase.

(3) Parameterized Distribution

In SDP, action generation is modeled as a Gaussian distribution in the latent action space, with the action distribution $\mu_{t}$ output by the backbone network and the standard deviation σ>0 as a learnable positive parameter.

(14) $$\pi_{\theta }\left(a_{t}^{s}|s_{t}\right)=N\left(\mu_{t},\mathrm{diag}\left(\sigma^{2}\right)\right)$$

where $\mu_{t}$: action distribution, which is output by the backbone network; σ: standard deviation, used to characterize the uncertainty of the policy.

Our method employs a learnable global parameter vector, assigning a shared standard deviation to each action dimension:

(15) $$\log\sigma =\log\left(s⊗1_{T}\right)$$

where ⊗: the broadcast replication operation; s: action space

The Gaussian distribution in the latent action space provides a stable continuous gradient path for loss function computation. This formulation models action uncertainty, accelerating convergence during the initial training phase while maintaining a smooth policy distribution within the latent policy space in subsequent training stages.

(4) Loss function

Apart from the Action Classification Head, the loss function of SDP consists of two additional parts:

Behavioral Cloning loss $L_{\mathrm{bc}}$:

(16) $$L_{\mathrm{bc}}={\left\|a_{t}-a_{t}^{s}\right\|}_{2}^{2}$$

To align the Student Distilled Policy with the Fused Attention Policy during distillation while minimizing cumulative errors, we introduce a behavioral cloning constraint based on Dataset Aggregation (DAgger) [49]. The behavioral cloning loss minimizes the discrepancy between the mean action output by the SDP and the FAP, allowing the student to gradually approximate the teacher’s behavior patterns in the policy space. This ensures consistency in basic movements. Meanwhile, DAgger’s data aggregation mechanism further reduces cumulative errors caused by distribution shifts through dynamic sampling of teacher trajectories and student behavior trajectories, thereby ensuring the stability and consistency of the distillation process.

Bidirectional KL divergence loss $L_{\mathrm{KL}}^{bi}$:

(17) $$L_{\mathrm{KL}}^{bi}=D_{KL}\left(\pi_{T}\|\pi_{S}\right)+D_{KL}\left(\pi_{S}\|\pi_{T}\right)$$

To enhance the distributional stability and generalization of the student policy within the latent action space, we introduce a bidirectional KL divergence loss during distillation. The forward KL divergence, $D_{KL}\left(\pi_{T}\|\pi_{S}\right)$, encourages the student to align its action distribution with high-probability regions of the teacher’s distribution in the latent action space, ensuring coverage of diverse behavioral modes. Conversely, the reverse KL divergence, $D_{KL}\left(\pi_{S}\|\pi_{T}\right)$, suppresses arbitrary dispersal of the student distribution into low-probability regions of the teacher, preventing distribution collapse and stabilizing exploration. This symmetric constraint mitigates instability from distributional variance mismatch while maintaining action diversity, enabling efficient distribution convergence during distillation.

## 4. Results

We conducted both sim-to-sim and sim-to-real validation for ToggleMimic. The testing device was a 23-DOF Unitree G1, with deployment on an i7 8th Gen CPU, and the control frequency set at 50 Hz. Our dataset is derived from a subset of the AMASS dataset, specifically the ACCAD and BMLmovi subsets, which were processed through redirection and filtering, encompassing a rich variety of motion samples with textual labels. We utilized an NVIDIA RTX 4090 GPU for training using IsaacGym Preview 4/IsaacSim 4.5.0 simulation environments. Additionally, to ensure safety, we first validated the policy’s transfer performance and robustness on the MuJoCo 3.1.0. To mitigate inference latency during model deployment and reduce the impact of sensor noise, we incorporated Domain Randomization and motor latency into the training process. Our evaluation metrics include the following: (1) Joint Position Tracking Error (JPTE), which measures the average deviation between the robot’s actual joint angles and the reference motion’s joint angles in radians (rad); (2) Success rate, which refers to the action execution success rate; (3) Generalization performance, referring to the model’s ability to generalize to unseen text; (4) Motion-Far Reset Ratio (MFRR), which represents the proportion of episodes that triggered resets due to the robot’s position deviating by 1.5 units from the dataset trajectory per unit training step. (5) Performance, Instantaneous scalar feedback corresponding to the system state at task termination events.

### 4.1. Validation of Action Switching and Execution Capability

To validate ToggleMimic’s ability to generate motions under multi-modal instruction conditions, we first conducted evaluations on a test set containing multiple types of actions. Our focus was on assessing the model’s execution accuracy and motion smoothness in response to different semantic instructions.

**Figure 4:**
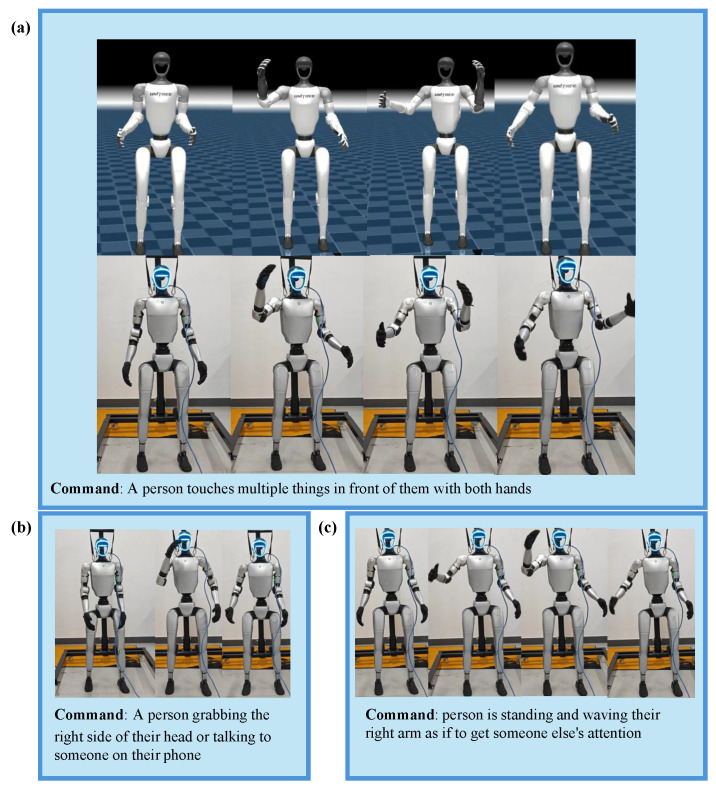
Real-World deployment. Our framework is capable of directly generating diverse actions from text commands and can be deployed in the real world. (**a**–**c**) Actions generated under three distinct text commands instructions demonstrate this framework’s zero-shot generalization capability for diverse language-action mappings.

As shown in Figure 4, the robot is able to successfully perform a variety of whole-body movements involving coordination between upper and lower limbs based on natural language instructions, including gestures. ToggleMimic can maintain a certain level of tracking accuracy and stability while switching between actions. In our method, the Joint Position Tracking Error is 1.07, with a success rate of 95%. Failures are mainly caused by misjudgments in similar motions (text commands) or phases. Through this experiment, we demonstrate the ability of our two-stage network, ToggleMimic, to generate diverse movements from text as well as its zero-shot sim-to-real transfer capability.

### 4.2. Generalization Ability

The precise generation of relevant motions from different linguistic expressions is a key capability for achieving natural human–computer interaction. We believe that the semantic-to-motion mapping learned through cross-modal attention, along with the dynamic adjustment capability introduced by the gating mechanism during the fusion stage, enables the model to capture the semantic association between motion and language in the latent space, thereby exhibiting a certain level of robustness to similar texts. To validate ToggleMimic’s generalization performance when faced with semantic variations and unseen instructions, we further tested the motion output of the student policy under several text instructions that are semantically similar but expressed differently.

**Figure 5:**
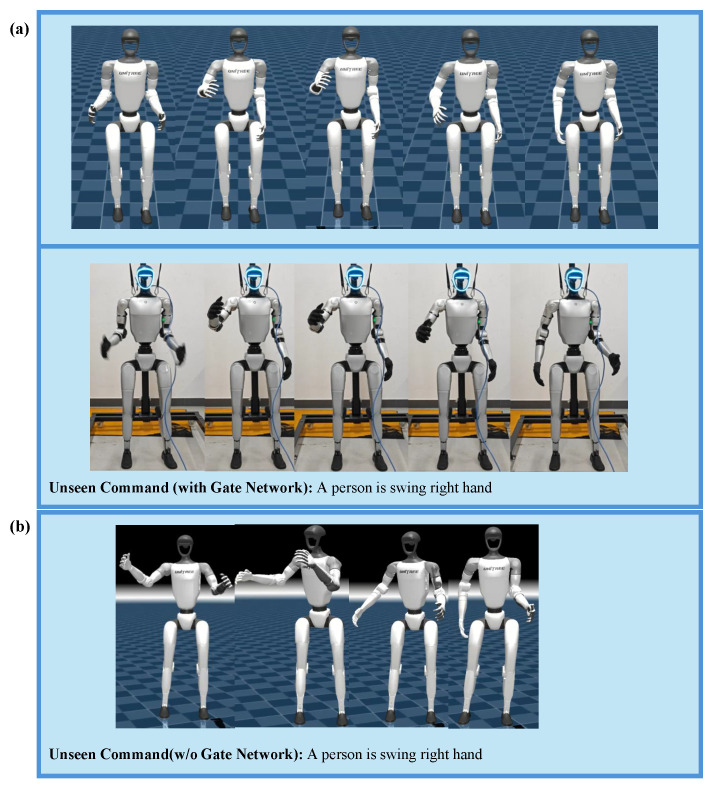
Generalization. Our framework possesses a certain level of generalization ability for unseen text, which is attributed to the cross-attention and gating mechanisms. (**a**) shows with Gate Network can successfully execute the unseen command; (**b**) shows without Gate Network fails to execute the unseen command.

As shown in Figure 5, ToggleMimic is still able to generate reasonable and semantically consistent motions for unseen text commands. To quantitatively verify this cross-lingual robustness, we analyzed the correlation between text semantic distance (based on cosine similarity in embedding space) and motion trajectory distance (L2 norm). We found a significant positive correlation between the two (Pearson r=0.352, p=0.008), indicating that motions generated from semantically similar instructions are also more alike. Further group analysis revealed that the motion differences within the same semantic group were significantly smaller than those between groups (1.890 vs. 2.586, shown in Table 3), with a group-level correlation reaching r=0.600 (p=0.039), confirming that the model effectively captures the semantic-motion correspondence in the latent space.

**Figure 6:**
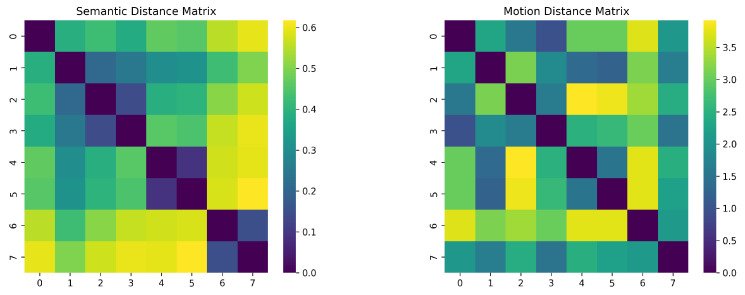
Semantic distance heatmap (**left**) motion distance heatmap (**right**).

To quantify the semantic-motion mapping relationship learned by the model, we calculated the semantic distances and motion trajectory distances for all pairs of instructions. As shown in Figure 6, low-value clusters formed by semantically proximate text correspond spatially to identical low-value regions in the motion distance heatmap. This structural correspondence indicates that the model has successfully mapped the proximity relationships in the semantic space to the motion generation space.

Our zero-shot generalization capability to unseen task descriptions arises from the computationally efficient LightCrossAttention module (LCA) and our Gating Network. The LCA enhances cross-modal feature alignment in the latent space, establishing rational semantic-to-action correspondences. Concurrently, the gating mechanism dynamically computes relevance weights between textual and motion features, enabling context-aware attention to semantic-relevant textual features during motion synthesis. This achieves precise semantic-action alignment.

### 4.3. Analysis of Efficiency, Interpretability, and Robustness

To validate the lightweight nature and interpretability of the proposed LightCrossAttention (LCA) module, we evaluate its computational cost and provide visualizations of the learned attention patterns. All experiments are conducted under identical hardware and model settings (NVIDIA RTX 4090, FP32 inference).

#### 4.3.1. Computational Efficiency

As shown in the Table 4, computational complexity analysis reveals a 55.09% reduction in FLOPs compared to standard Transformer through our optimized attention mechanism, which streamlines computation paths and eliminates redundant operations. LCA delivers 1.28× inference speedup under identical hardware conditions. This acceleration manifests not only from theoretical complexity reduction but also from optimized memory access patterns and enhanced computational parallelism. Crucially, latency improvements will directly translate to enhanced user experience and operational feasibility.

#### 4.3.2. Attention Heatmap Visualization

**Figure 7:**
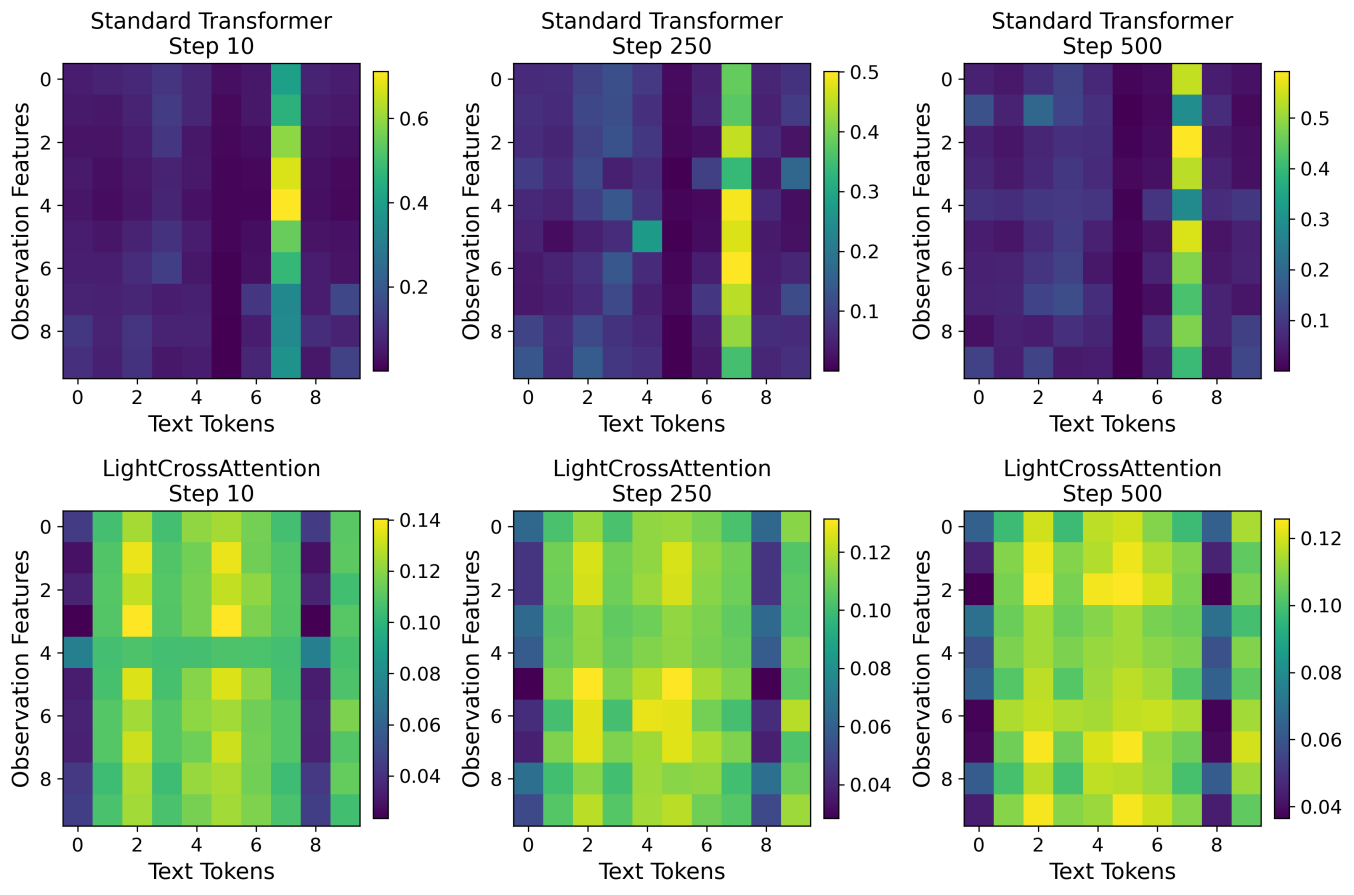
Attention Heatmaps. Comparative attention heatmaps of standard Transformers (**top row**) and LightCrossAttention (**bottom row**).

To evaluate interpretability, we visualize attention heatmaps between textual semantic tokens and action dimensions. Representative results are shown in Figure 7.

The attention pattern comparison reveals fundamentally divergent strategies between standard Transformers and LightCrossAttention (LCA). As illustrated in Figure 7, standard Transformers exhibit a relatively concentrated yet sparse attention distribution—termed the “precision-focused” strategy. This pattern demonstrates how standard Transformers selectively attend to critical features via complex gating mechanisms while suppressing numerous irrelevant connections.

In contrast, LCA employs a uniform and dense attention distribution through its broad-spectrum perception strategy. This design enables global contextual awareness by maintaining active connections across the feature space, comprehensively integrating semantic information. This approach is particularly effective for our task domain, where textual instructions are semantically dense—each token carries meaningful information for task execution without superfluous content. LCA’s uniform weight distribution fully utilizes this characteristic, ensuring textual and observational features contribute to the representation while avoiding information loss from aggressive feature selection. By eliminating complex selection processes while preserving global context, LCA achieves computational efficiency without compromising representation quality.

#### 4.3.3. Robustness Under Partial Perception Occlusions

To evaluate the robustness of this strategy in real-world scenarios where sensor information may be missing or corrupted, we introduce controlled perceptual occlusion during the evaluation process. Specifically, for a given ratio, a proportion *p* (p∈[0,0.2]) of the observation dimensions will be randomly set to zero, simulating partial sensor failure or occlusion in proprioceptive channels.The results are summarized in the table below, where we report two complementary metrics:

L2 Distance: measures the absolute deviation in the action space.

(18) $$L2\left(a_{\mathrm{occ}},a_{\mathrm{base}}\right)=∥a_{\mathrm{occ}}-a_{\mathrm{base}}∥$$

Cosine Similarity: the directional consistency between actions

(19) $$\mathrm{Cosine}\left(a_{\mathrm{occ}},a_{\mathrm{base}}\right)=\frac{a_{\mathrm{occ}}\cdot a_{\mathrm{base}}}{∥a_{\mathrm{occ}}∥\cdot ∥a_{\mathrm{base}}∥}$$

where $a_{\mathrm{occ}}$: the action generated under occlusion, $a_{\mathrm{base}}$: the action generated under fully available observations.

As shown in Table 5, experimental results reveal an exponential-decay-like degradation pattern. Under mild occlusion (5–10%), action deviation remains low while directional consistency stays high (>0.904), demonstrating the system’s robust operational capability. As occlusion intensifies, action deviation increases—but the policy maintains reasonable directional consistency (Cosine > 0.6 even at 20% occlusion)—quantitatively confirming the strategy’s strong robustness to incomplete perception data.

### 4.4. Ablation Study

To systematically evaluate the contributions of each key module and loss term to the model’s performance, we conducted ablation studies on both the Fused Attention Policy and the Student Distilled Policy. Each ablation model was trained for 10,000 epochs (RTX 4090 GPU, with each training session taking approximately 10 h), and the performance metrics were averaged over the last 1000 epochs (from epoch 9000 to 10,000) to mitigate the impact of random fluctuations on the evaluation. All experiments were independently repeated three times with different random seeds, and the data collected from each run were averaged to eliminate biases caused by random initialization.

#### 4.4.1. Fused Attention Policy

(1) Module Ablation

We ablated the Early Fusion module, the LSTM module in the μ module, and the LCA module to quantify their impact on temporal dependency modeling and cross-modal interaction performance.

As shown in Table 6, after removing the EA layer, although JPTE decreased by 0.24 compared to the baseline and reached the optimal value, the success rate relatively dropped by 7%. Removing the LCA module caused training divergence, indicating that the LCA module plays a crucial role in establishing differentiated semantic alignment features and is a foundational condition for stable policy learning. The JPTE of our complete model decreased by 0.15 compared to the baseline, but its success rate was the highest (95%), demonstrating that our complete model strikes a balance between success rate and JPTE and is thus the optimal model.

(2) Reward Function Sensitivity Analysis

To validate the design rationale of the compositional reward function and dissect the contributions of each component to learning stability and tracking accuracy, we conducted a systematic sensitivity analysis following ablation study methodology. This analysis rigorously assesses the validity of component selection and weighting configurations, as misconfigured reward functions inherently induce training instability or suboptimal policies.

We conducted a series of ablation studies systematically removing specific reward components from the complete compositional reward function. Ablation groups are defined as follows (Table 7).

1. Control Penalties Are the Cornerstone of Learning Stability.
  Removing torque regularization (Remove Control Penalties) or motor torque limits (Remove Limits) causes significant performance declines—MFRR deteriorates by 19% and 10% respectively (Table 8). Analysis reveals that such removals induce frequent violations of motor torque limits, driving the agent toward aggressive policies. While these policies yield short-term gains, they destabilize learning dynamics and ultimately constrain long-term performance. This confirms torque penalties are critical for preventing policy divergence, ensuring stable learning, and guaranteeing system safety.
2. Tracking Rewards Exhibit Cumulative Effects Across Motion Control.
  Eliminating individual tracking components (joint, orientation, or position) increases MFRR by 6–9%. Removing constraint terms further destabilizes the system, proving the polynomial structure is not arbitrary but necessary for coordinated control. Crucially, in our task, excessive deviation from the reference trajectory triggers early environment reset, implying the agent receives dual guidance: explicit tracking rewards and implicit feedback from termination conditions.
  We believe even without specific tracking terms (e.g., joint/velocity trackings), the agent learns basic motion direction through repeated failures and resets, minimizing premature termination. This explains the modest 6–9% performance degradation: the agent develops a fundamental motion strategy but lacks continuous explicit supervision for trajectory refinement, motion coherence, and action fidelity. Thus, tracking rewards primarily enhance precision, continuity, and execution fidelity—not the generation of baseline motion capability.

#### 4.4.2. Student Distilled Policy

(1) Sensitivity Analysis of Network Components

We separately remove behavior cloning loss (BC loss), KL divergence loss (KL loss), and Action Classification Head to verify the contributions of BC loss, KL loss, and Action Classification Head to the model.

As shown in Table 9, the model training under the No-KL configuration fails to converge, confirming the KL divergence loss is essential for aligning student and teacher policy distributions. In the No-ACH configuration, the MFRR in the early stage of training increases by 6% compared with the baseline, showing obvious tracking and training instability. This suggests that the classification auxiliary task can help the model quickly establish semantic alignment of action categories in the early stage. The trajectory tracking accuracy of the No-BC configuration decreases by 0.14 compared with the complete model, indicating that the behavior cloning term has a significant effect in guiding the student policy to perform low-level action imitation. Overall, jointly optimizing BC loss, KL loss, and Action Classification Head is necessary for achieving stable and efficient policy distillation.

(2) Sensitivity Analysis of Loss Weights

To validate the rationale behind the weight design in the loss function during policy distillation, we systematically analyzed how the weighting coefficients of the behavioral cloning (BC) loss $L_{\mathrm{BC}}$ and bidirectional KL divergence loss $L_{\mathrm{KL}}^{\mathrm{bi}}$ influence distillation performance. The total loss function is defined as follows:

(20) $$L_{\mathrm{total}}=\lambda_{\mathrm{BC}}\cdot L_{\mathrm{BC}}+\lambda_{\mathrm{KL}}\cdot L_{\mathrm{KL}}^{\mathrm{bi}}$$

where $\lambda_{\mathrm{BC}}$ and $\lambda_{\mathrm{KL}}$ are the weights corresponding to their respective loss functions. All other hyperparameters were fixed and trained across eight distinct weight configurations.

**Figure 8:**
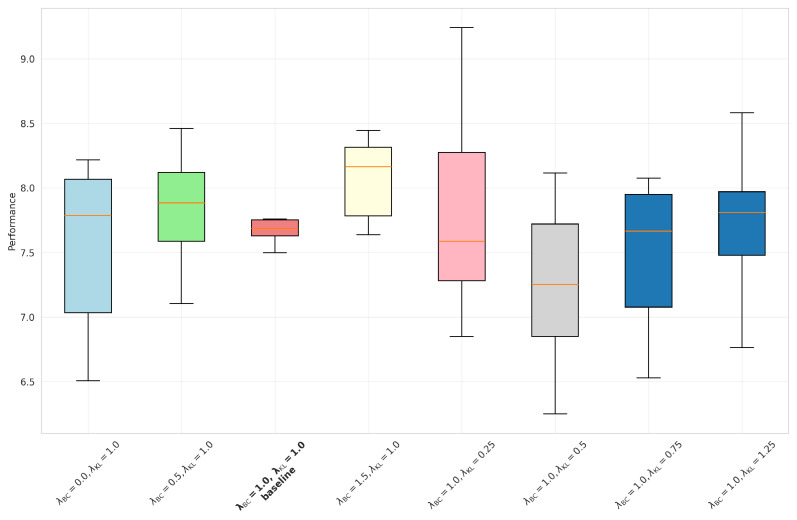
Boxplot of final reward across BC–KL weight combinations.

Based on the Table 10 and boxplot analysis Figure 8, the configuration of loss weights significantly impacts both model performance and training stability. When $\lambda_{KL}$ is fixed at 1.0, increasing $\lambda_{BC}$ elevates performance while reducing BC loss; however, excessive weight ($\lambda_{BC}=1.5$) elevates performance variance (0.66), with boxplots indicating greater volatility than baseline—signifying degraded training stability. Conversely, with $\lambda_{BC}$ fixed at 1.0, raising $\lambda_{KL}$ effectively enhances stability (performance std decreasing from 0.70 to 0.47), though overly stringent constraints ($\lambda_{KL}=1.25$) suppress performance, reducing performance. In summary, adopting $\lambda_{BC}=1.0$ and $\lambda_{KL}=1.0$ as the default configuration achieves optimal tradeoffs across rewards (7.82), stability (std of 0.51), and loss metrics, with boxplot analysis Figure 8 further corroborating this stability through the most compact box distribution.

(3) Distillation Stability Validation

**Figure 9:**
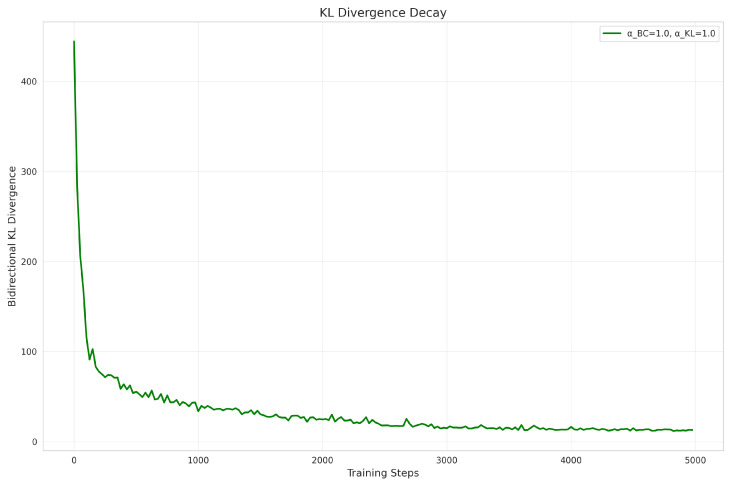
KL divergence decay.

To demonstrate the convergence behavior of policy distillation, we recorded the evolution of bidirectional KL divergence during training under the default configuration ($\lambda_{BC}=1.0$, $\lambda_{KL}=1.0$). As shown in Figure 9, the KL divergence exhibits a monotonic decreasing trend with training steps, smoothly converging from a high initial value to a final value of 12.83 without severe oscillations throughout training. This confirms the excellent training stability and convergence fidelity of our distillation method.

## 5. Discussion

### 5.1. Contributions

Our ToggleMimic framework proposed in this study has achieved a new breakthrough in text-driven motion generation tasks. Unlike previous strategies that relied on a single modality or static mapping, this method introduces a teacher–student two-stage distillation mechanism, which realizes high-precision guidance from the teacher policy FAP to the lightweight execution of the student policy SDP, significantly improving the model’s stability and generalization under complex instructions. This framework establishes a unified optimization objective between language understanding and motion generation, enabling the robot not only to accurately perform actions corresponding to known texts but also to generate reasonable motion sequences when facing unseen instructions. Experimental results show that ToggleMimic outperforms traditional end-to-end imitation learning methods in diverse action execution and cross-instruction generalization tasks, verifying its effectiveness and scalability in the field of cross-modal understanding and language- driven control.

### 5.2. Limitation

Although this method shows some advantages in generalization and stability, it still has several limitations:

(1) Compute constraints and network capacity

Since the current training is mainly performed on a single RTX 4090 GPU, the model can only achieve stable convergence on dozens of instruction labels. In our experiments, further increasing the text categories or lengthening the action sequence led to significant VRAM pressure and risk of overfitting. Future work could consider incorporating models that can learn latent vectors, such as VAEs [44], for training, or utilizing lightweight techniques like parameter sharing, model pruning, or low-rank adaptation to reduce computational requirements, thereby supporting larger-scale text-to-action generation learning.

(2) Lack of external perception information

This research focuses on the generation task from language to action, without using external perceptual information from vision or point cloud sensors for processing, which limits the robot’s behavioral ability in complex environments (such as obstacle crossing, obstacle avoidance, or multi-agent interaction scenarios) to a certain extent. In the future, multimodal perception systems combined with text can be utilized to achieve unified modeling from language to environmental perception and then to action decision-making.

### 5.3. Implications for Future Humanoid Robot Design

Our method offers several implications for the development of next-generation humanoid systems. The proposed lightweight cross-modal architecture reduces inference cost, enabling language-conditioned control to run in real time on edge computing hardware without cloud dependence. The explicit attention alignment between instructions and joint motions improves interpretability, supporting safer deployment and more efficient debugging. Moreover, the ability to switch behaviors based solely on changing task descriptions demonstrates strong potential for real-world multi-task autonomy in household, service, and industrial settings.

### 5.4. Future Work

Our current ToggleMimic framework establishes an effective, end-to-end paradigm for generating actions directly from text, eliminating the need for pre-generated trajectories or complex planning modules. Building upon this foundation, several promising directions warrant further investigation to advance towards more general, adaptive, and robust humanoid control.

#### 5.4.1. Multilingual Language Adaptation

ToggleMimic focuses on textual commands to establish a scalable and baseline framework for agent instruction-following. We concur that supporting multilingual instructions constitutes a promising avenue for enhancing the general-purpose capability of embodied AI agents. Extending systems to robustly comprehend and execute multilingual directives entails substantial challenges—including data curation, language model integration, and cross-lingual grounding—which we identify as requiring dedicated research investment. The next step is to focus on exploring multilingual instruction adaptation mechanisms and studying how to map specific actions to diverse linguistic expressions.

#### 5.4.2. Semantic Evolution

We plan to transcend the current reliance on pre-trained text embeddings by exploring methods to directly obtain action-relevant features through deeper attention mechanisms. More significantly, a paramount next step is the integration of visual and auditory inputs alongside text commands. By incorporating vision-language-action (VLA) models [50,51,52,53] or audio processing networks, we can encode visual scenes and sound cues into a shared latent space with proprioception and language. This would enable true context-aware operation, allowing the robot to execute commands like “pick up the red cup on the table” (vision) or “move towards the ringing phone” (audio), dramatically increasing robustness in unstructured environments.

#### 5.4.3. Longitudinal Evaluation and Lifelong Learning

To further enhance the practical utility of ToggleMimic, a critical next step is to investigate its long-term adaptation capabilities. Future work will focus on enabling lifelong learning, allowing the system to incrementally acquire new language commands without forgetting previous skills, and to adapt its behavior through prolonged interaction with users. This will involve developing safe online fine-tuning algorithms and conducting longitudinal studies to assess performance evolution and user interaction over time. 

## Figures and Tables

**Table 1:** Observation.

State	Dim
Text Embedding	512
Root angular velocity	3
Root projected gravity	3
Joint pos	23
Joint vel	23
Actions	23
Reference motion phase	1
Body position difference	81
Reference position pos	81

**Table 2:** Reward function.

Reward	Expression	Weight
Full-Body Position Tracking	$\exp\left(-\frac{∥p-p^{\mathrm{ref}}{∥}_{2}^{2}}{\sigma }\right)$	1.0
Key Position Tracking	$\exp\left(-\frac{∥p_{k}-p_{k}^{\mathrm{ref}}{∥}_{2}^{2}}{\sigma_{k}}\right)$	1.6
Foot Position Tracking	$\exp\left(-\frac{∥p_{\mathrm{feet}}-p_{\mathrm{feet}}^{\mathrm{ref}}{∥}_{2}^{2}}{\sigma_{\mathrm{feet}}}\right)$	2.1
Full-Body Orientation Tracking	$\exp\left(-\frac{∥\theta_{\mathrm{rot}}-\theta_{\mathrm{rot}}^{\mathrm{ref}}{∥}_{2}^{2}}{\sigma_{\mathrm{rot}}}\right)$	0.5
Full-Body Angular Velocity Tracking	$\exp\left(-\frac{∥\omega_{\mathrm{body}}-\omega_{\mathrm{body}}^{\mathrm{ref}}{∥}_{2}^{2}}{\sigma_{\omega }}\right)$	0.5
Full-Body Linear Velocity Tracking	$\exp\left(-\frac{∥v_{\mathrm{body}}-v_{\mathrm{body}}^{\mathrm{ref}}{∥}_{2}^{2}}{\sigma_{v}}\right)$	0.5
Joint Position Tracking	$\exp\left(-\frac{∥q-q^{\mathrm{ref}}{∥}_{2}^{2}}{\sigma_{q}}\right)$	0.75
Joint Velocity Tracking	$\exp\left(-\frac{∥\dot{q}-{\dot{q}}^{\mathrm{ref}}{∥}_{2}^{2}}{\sigma_{\dot{q}}}\right)$	0.5
Torque Penalty	${∥\tau ∥}_{2}^{2}$	−0.000001
Action Smoothness Penalty	$∥a_{t}-a_{t-1}{∥}_{2}^{2}$	−0.5
Foot Slippage Penalty	$\sum_{i\in \mathrm{feet}}∥v_{\mathrm{foot},i}{∥}_{2}\cdot I(∥F_{\mathrm{contact},i}{∥}_{2}>\epsilon_{F})$	−1.0
Joint Position Limits	$\sum_{i=1}^{N_{q}}\left[-{\left(q_{i}-q_{i,\mathrm{low}}^{\mathrm{soft}}\right)}_{+}+{\left(q_{i}-q_{i,\mathrm{high}}^{\mathrm{soft}}\right)}_{+}\right]$	−10.0
Joint Velocity Limits	$\sum_{i=1}^{N_{q}}{\left(|{\dot{q}}_{i}|-{\dot{q}}_{i}^{\mathrm{soft}}\right)}_{+}^{\mathrm{clip}}$	−5.0
Torque Limits	$\sum_{i=1}^{N_{q}}{\left(|\tau_{i}|-\tau_{i}^{\mathrm{soft}}\right)}_{+}$	−5.0
Termination	I(reset)·I(¬timeout)	−200.0

Notations: *p*/*v*/ω: position, velocity, angular velocity, $F_{\mathrm{contact}}$: contact force, *q*/$\dot{q}$: joint position/velocity, τ: torque, $a_{t}$: actions; $\epsilon_{F}$: foot-sliding threshold; σ*: tracking sigma, $N_{q}$: number of joints; $\cdot^{\mathrm{soft}}$: soft limits.

**Table 3:** Intra- vs. Inter-group distance comparison.

Metric	Intra-Pair Mean	Inter-Pair Mean
Semantic Distance	0.193	0.465
Motion Trajectory Similarity	1.890	2.586

**Table 4:** Comparison between standard transformer and LightCrossAttention (ours). ↓ indicates the reduction of FLOPs.

Metrics	Standard Transformer	LightCrossAttention (Ours)	Improvement
FLOPs	1,195,520	**536,960**	↓ 55.09 %
Latency per step	0.599 ± 0.126 ms	**0.468 ± 0.062** ms	1.28× faster

**Table 5:** Comparison of occlusion ratio, L2 distance, and cosine similarity. The arrows ↓ and ↑ indicate that lower and higher values are better, respectively.

Occlusion Ratio	L2 Distance ↓	Cosine Similarity ↑
0%	0.000	1.000
5%	2.875	0.894
10%	2.874	0.904
15%	4.161	0.825
20%	5.457	0.617

**Table 6:** Ablation study on FAP.

Metric	JPTE	Success Rate (%)	MFRR (%)
Full	1.07	95	11
No-EA	0.98	82	12
No-LSTM (baseline)	1.22	93	12
No-LCA	X	X	83

Notations: No-EA: The Early Fusion layer is removed, and the observation is directly concatenated with the text embeding; No-LSTM: μ module w/o LSTM; No-LCA: The LCA is replaced with a 3-layer, 1024-dimensional MLP.

**Table 7:** Reward components.

Group	Component
Remove Position Tracking	Full-Body Position Tracking
	Key Position Tracking
	Foot Position Tracking
Remove Orientation Velocity Tracking	Full-Body Orientation Tracking
	Full-Body Angular Velocity Tracking
	Full-Body Linear Velocity Tracking
Remove Joint Tracking	Joint Position Tracking
	Joint Velocity Tracking
Remove Control Penalties	Torque Penalty
	Action Smoothness Penalty
	Foot Slippage Penalty
Remove Limits	Joint Position Limits
	Joint Velocity Limits
	Torque Limits
	Termination

**Table 8:** Comparison of reward settings in terms of JPTE and MFRR.

Reward Setting	JPTE	MFRR (%)
Remove Control Penalties	2.89	30
Remove Limits	1.45	21
Remove Orientation Velocity Tracking	1.31	17
Remove Position Tracking	1.35	19
Remove Joint Tracking	1.29	17
Baseline (full reward)	1.07	11

**Table 9:** Ablation study on SDP.

Metric	JPTE	Success Rate (%)	MFRR (%) (<3000 Epochs)
FULL (baseline)	0.85	94	11
No-BC	0.98	93	13
No-KL	X	X	X
No-ACH	0.89	94	17

Notations: No-BC: The behavior cloning loss is removed; No-KL: the KL divergence loss is removed; No-ACH: the Action Classification Head and its loss is removed.

**Table 10:** Final performance and loss under different BC and KL weights. The baseline setting is BC = 1.0, KL = 1.0.

BC Weight	KL Weight	Final Performance	Final BC Loss	Final KL Loss
0.0	1.00	7.56 ± 0.58	0.0361	13.68
0.5	1.00	7.84 ± 0.42	0.0363	13.79
1.0	0.25	7.77 ± 0.70	0.0347	13.17
1.0	0.50	7.23 ± 0.59	0.0373	14.15
1.0	0.75	7.47 ± 0.56	0.0351	13.30
1.0	1.00	7.82 ± 0.51	0.0338	12.83
1.0	1.25	7.76 ± 0.47	0.0333	12.69
1.5	1.00	8.07 ± 0.66	0.0310	11.86

## Data Availability

The original contributions presented in this study are included in the article. Further inquiries can be directed to the corresponding author.
